# Supplementary material for: Head-to-head comparison of diagnostic accuracy of TB screening tests: Chest-X-ray, Xpert TB host response, and C-reactive protein
Source: medRxiv. 2024 Jun 21:2024.06.20.24308402. Preprint. [Version 1] doi: 10.1101/2024.06.20.24308402 (PMC11213098; doi:10.1101/2024.06.20.24308402)
Supplement: Supplement 1 [file media-1.pdf]

Additional contributors from the R2D2 TB Network

First name	Surname	Affiliation
**India**		
Shanmugasundaram	Elango	Christian Medical College, Vellore, India
Jerusha	Emmanuel	Christian Medical College, Vellore, India
Vinita	Ernest	Christian Medical College, Vellore, India
Priyadarshini	Gajendran	Christian Medical College, Vellore, India
Flavita	John	Christian Medical College, Vellore, India
Bharath	Karthikeyan	Christian Medical College, Vellore, India
Divya	Mangal	Christian Medical College, Vellore, India
Swetha	Sankar	Christian Medical College, Vellore, India
Rajasekar	Sekar	Christian Medical College, Vellore, India
Reena	Sekar	Christian Medical College, Vellore, India
Deepa	Shankar	Christian Medical College, Vellore, India
Mary	Shibiya	Christian Medical College, Vellore, India
Sai	Vijayasree	Christian Medical College, Vellore, India
**Philippines**		
Jared	Almonte	De La Salle Medical and Health Sciences Institute, Cavite
Kevin Joshua	Alonzo	National TB Reference Laboratory, Research Institute for Tropical Medicine, Department of Health
Mary Faith	Angcaya	De La Salle Medical and Health Sciences Institute, Cavite
Joseph Edwin L.	Bascuña	National TB Reference Laboratory, Research Institute for Tropical Medicine, Department of Health
Ramon P.	Basilio	National TB Reference Laboratory, Research Institute for Tropical Medicine, Department of Health
Asella Ruvijean	Cariaga	De La Salle Medical and Health Sciences Institute, Cavite, Philippines
Gabriella	Castillon	De La Salle Medical and Health Sciences Institute, Cavite
Karlo	Dayawon	De La Salle Medical and Health Sciences Institute, Cavite
Raul	Destura	National Institutes of Health, University of the Philippines Manila
Jezreel	Esguerra	De La Salle Medical and Health Sciences Institute, Cavite
Eleonor	Garcia	De La Salle Medical and Health Sciences Institute, Cavite
Darecil	Gelina	De La Salle Medical and Health Sciences Institute, Cavite
Joseph Aldwin	Goleña	De La Salle Medical and Health Sciences Institute, Cavite
Maria Marissa	Golla	De La Salle Medical and Health Sciences Institute, Cavite
Emmanuelle	Gutierrez	De La Salle Medical and Health Sciences Institute, Cavite
Gidalthi Jonathan	Ilagan	De La Salle Medical and Health Sciences Institute, Cavite
Dodge R.	Lim	National TB Reference Laboratory, Research Institute for Tropical Medicine, Department of Health
Jaiem	Maranan	De La Salle Medical and Health Sciences Institute, Cavite
Danaida	Marcelo	De La Salle Medical and Health Sciences Institute, Cavite
Leonedy	Masangcay	De La Salle Medical and Health Sciences Institute, Cavite
Jenkin	Mendoza	National TB Reference Laboratory, Research Institute for Tropical Medicine, Department of Health
Angelita	Pabruada	De La Salle Medical and Health Sciences Institute, Cavite
Laarean	Perlas	De La Salle Medical and Health Sciences Institute, Cavite
Annalyn	Reyes	De La Salle Medical and Health Sciences Institute, Cavite
Roeus Vincent Arjay G.	Reyes	National TB Reference Laboratory, Research Institute for Tropical Medicine, Department of Health

First name	Surname	Affiliation
Lorenzo	Reyes	National TB Reference Laboratory, Research Institute for Tropical Medicine, Department of Health
Maria Guileane	Sanchez-Pogosa	National TB Reference Laboratory, Research Institute for Tropical Medicine, Department of Health
Maricef	Tonquin	De La Salle Medical and Health Sciences Institute, Cavite
**South Africa**		
Shima	Abdulgadar	Stellenbosch University, Cape Town, South Africa
Cammy	Botha	Stellenbosch University, Cape Town, South Africa
Brigitta	Derendinger	Stellenbosch University, Cape Town, South Africa
Jane	Fortuin	Stellenbosch University, Cape Town, South Africa
Siphosethu	Gonya	Stellenbosch University, Cape Town, South Africa
Chumani	Hatile	Stellenbosch University, Cape Town, South Africa
Megan	Hendrikse	Stellenbosch University, Cape Town, South Africa
Charlotte	Lawn	Stellenbosch University, Cape Town, South Africa
Disha	Mathoorah	Stellenbosch University, Cape Town, South Africa
Desiree Lem	Mbu	Stellenbosch University, Cape Town, South Africa
Zintle	Ntetha	Stellenbosch University, Cape Town, South Africa
Anna	Okunola	Stellenbosch University, Cape Town, South Africa
Zaida	Palmer	Stellenbosch University, Cape Town, South Africa
Fikiswa	Seti	Stellenbosch University, Cape Town, South Africa
Charmaine	Van Der Walt	Stellenbosch University, Cape Town, South Africa
Lusanda	Yekani	Stellenbosch University, Cape Town, South Africa
**Uganda**		
chriLucy	Asege	Walimu, Kampala, Uganda
Alice	Bukirwa	Walimu, Kampala, Uganda
David	Katumba	Walimu, Kampala, Uganda
Esther	Kisakye	Walimu, Kampala, Uganda
Wilson	Mangeni	Walimu, Kampala, Uganda
Job	Mukwatamundu	Walimu, Kampala, Uganda
Sandra	Mwebe	Walimu, Kampala, Uganda
Annet	Nakaweesa	Walimu, Kampala, Uganda
Martha	Nakaye	Walimu, Kampala, Uganda
Talemwa	Nalugwa	Walimu, Kampala, Uganda
Irene	Nassuna	Walimu, Kampala, Uganda
Irene	Nekesa	Walimu, Kampala, Uganda
Justine	Nyawere	Walimu, Kampala, Uganda
John Baptist	Ssonko	Walimu, Kampala, Uganda
**Vietnam**		
Hai	Dang	Vietnam National Tuberculosis Program-University of California San Francisco Research Collaboration Unit; Center for Promotion of Advancement of Society, Hanoi, Vietnam
Luong	Dinh	Vietnam National Lung Hospital
Hang	Do	Hanoi Lung Hospital, Hanoi, Vietnam
Tam	Do	Hanoi Lung Hospital, Hanoi, Vietnam
Thuong	Do	Vietnam National Lung Hospital
Dung	Dao	Hanoi Lung Hospital, Hanoi, Vietnam
Ha	Doan	National TB reference Lab/ Vietnam National Lung Hospital, Hanoi, Vietnam

First name	Surname	Affiliation
Thien	Doan	Hanoi Lung Hospital, Hanoi, Vietnam
Huy	Ha	Vietnam National Tuberculosis Program-University of California San Francisco Research Collaboration Unit, Center for Promotion of Advancement of Society, Hanoi, Vietnam
Oanh	Lai	Hanoi Lung Hospital, Hanoi, Vietnam
Hien	Le	Vietnam National Tuberculosis Program-University of California San Francisco Research Collaboration Unit; Center for Promotion of Advancement of Society, Hanoi, Vietnam
Nguyet	Le	National TB reference Lab/ Vietnam National Lung Hospital, Hanoi, Vietnam
Anh	Nguyen	Hanoi Lung Hospital, Hanoi, Vietnam
Hanh	Nguyen	Vietnam National Tuberculosis Program-University of California San Francisco Research Collaboration Unit; Center for Promotion of Advancement of Society, Hanoi, Vietnam
Hoa	Nguyen	Vietnam National Lung Hospital
Hoang	Nguyen	Hanoi Lung Hospital, Hanoi, Vietnam
Thanh	Nguyen	Hanoi Lung Hospital, Hanoi, Vietnam
Yen	Nguyen	Hanoi Lung Hospital, Hanoi, Vietnam
Ha	Phan	Vietnam National Tuberculosis Program-University of California San Francisco Research Collaboration Unit, Center for Promotion of Advancement of Society, Hanoi, Vietnam
Nam	Pham	Vietnam National Tuberculosis Program-University of California San Francisco Research Collaboration Unit, Hanoi Lung Hospital, Hanoi, Vietnam
Thuong	Pham	Hanoi Lung Hospital, Hanoi, Vietnam
Trang	Trinh	Vietnam National Tuberculosis Program-University of California San Francisco Research Collaboration Unit, Center for Promotion of Advancement of Society, Hanoi, Vietnam
Phuong	Vu	Hanoi Lung Hospital, Hanoi, Vietnam
Trung	Vu	National TB reference Lab/ Vietnam National Lung Hospital, Hanoi, Vietnam
**USA**		
Robert	Castro	University of California San Francisco, San Francisco, CA, USA
Adithya	Cattamanchi	University of California Irvine, Irvine, CA, USA
Catherine	Cook	University of California San Francisco, San Francisco, CA, USA
Sophie	Huddart	University of California San Francisco, San Francisco, CA, USA
Devan	Jaganath	University of California San Francisco, San Francisco, CA, USA
Midori	Kato-Maeda	University of California San Francisco, San Francisco, CA, USA
Tessa	Mochizuki	University of California San Francisco, San Francisco, CA, USA
Ruvandhi	Nathavitharana	Beth Israel Deaconess Medical Center, Harvard Medical School, Boston, MA, USA
Payam	Nahid	University of California San Francisco, San Francisco, CA, USA
Kevin	Nolan	University of California San Francisco, San Francisco, CA, USA
Kinari	Shah	University of California San Francisco, San Francisco, CA, USA
Swati	Sudarsan	University of California San Francisco, San Francisco, CA, USA
Christina	Yoon	University of California San Francisco, San Francisco, CA, USA
**Germany**		
Maria del Mar	Castro Noriega	Heidelberg University Hospital, Heidelberg, Germany

First name	Surname	Affiliation
Theresa	Pfurtscheller	Heidelberg University Hospital, Heidelberg, Germany
Seda	Yerlikaya	Heidelberg University Hospital, Heidelberg, Germany
**Switzerland**		
Matthew	Arentz	FIND, Geneva, Switzerland
Nathalie	Frey	FIND, Geneva, Switzerland
Sam	Linsen	FIND, Geneva, Switzerland
