# Supplementary material for: Head-to-head comparison of diagnostic accuracy of TB screening tests: Chest-X-ray, Xpert TB host response, and C-reactive protein
Source: medRxiv. 2024 Jun 21:2024.06.20.24308402. Preprint. [Version 1] doi: 10.1101/2024.06.20.24308402 (PMC11213098; doi:10.1101/2024.06.20.24308402)
Supplement: Supplement 2 [file media-2.pdf]

APPENDIX

Table S1. R2D2 TB Network study enrolment sites and ethics committees 2
Figure S1. Sequential testing algorithms. 3
Table S2. Summary of index test results by reference standard classification 3
Table S3. Results using subgroup-specific cut-points 4
Table S4. Positive predictive value and negative predictive value in a hypothetical cohort of 1,000 with 10% TB prevalence 5
Table S5. The potential number of sputum tests averted by using a triage test in a hypothetical cohort of 1000 with 10% TB prevalence 6
Figure S2. Sputum Xpert reference standard receiver operating characteristic curve...........7
Figure S3. Selection of cut points for two-step screening algorithm combining CRP and CAD4TB. 8
Table S6. Head-to-head comparison of diagnostic accuracy against sputum Xpert reference standard. 10
Figure S5. Subgroup analysis of two-step screening algorithm Xpert HR-CAD4TB using a sequential negative serial screening algorithm. 11
Figure S6. Subgroup analysis of two-step screening algorithm CRP-CAD4TB using a sequential negative serial screening algorithm. 12
Figure S7. Subgroup analysis of two-step screening algorithm CRP-Xpert HR using a sequential negative serial screening algorithm. 13
Figure S8. Subgroup analysis of two-step screening algorithm Xpert HR-CAD using a sequential negative serial screening algorithm. 14
Table S7. Agreement between binary triage tests (percent agreement, Cohen's Kappa statistic) 15

Table S1. R2D2 TB Network study enrolment sites and ethics committees

City, Country	Enrollment sites	Ethics Committee*
Vellore, India	CMC Pulmonary Outpatient Department, Primary care clinics in Vellore (Shalom/LCC, Chittor, CHAD) and Chitoor (CMC satellite campus)	Christian Medical College Institutional Review Board (13256)
Hanoi, Vietnam	Outpatient departments, Hanoi Lung Hospital	Ministry of Health Ethical Committee for National Biological Medical Research (94/CN-HĐĐĐ); National Lung Hospital Ethical Committee for Biological Medical Research (566/2020/NCKH); Hanoi Lung Hospital Science and Technology Initiative Committee (22/BVPHN)
Dasmariñas City, Philippines	Community-based screening in Dasmariñas City and nearby municipalities, outpatient clinics in Dasmariñas City	De La Salle Health Sciences Institute Independent Ethics Committee (2020-33-02-A)
Cape Town, South Africa	Scottsdene and Wallacedene primary care clinics; Brooklyn Chest Hospital; Khayelitsha District Health Center; Kraaifontein Community Health Clinic	Stellenbosch University Health Research Ethics Committee (M20/07/020)
Kampala, Uganda	Mulago Outpatient Department, Kisenyi Health Center,	Makerere University, College of Health Sciences, School of Medicine, Research Ethics Committee (2020-182)

* The study was additionally approved by the University of California San Francisco Institutional Review Board (20-32670), and the University of Heidelberg Ethics Committee of the Medical Faculty (S-539/2020)

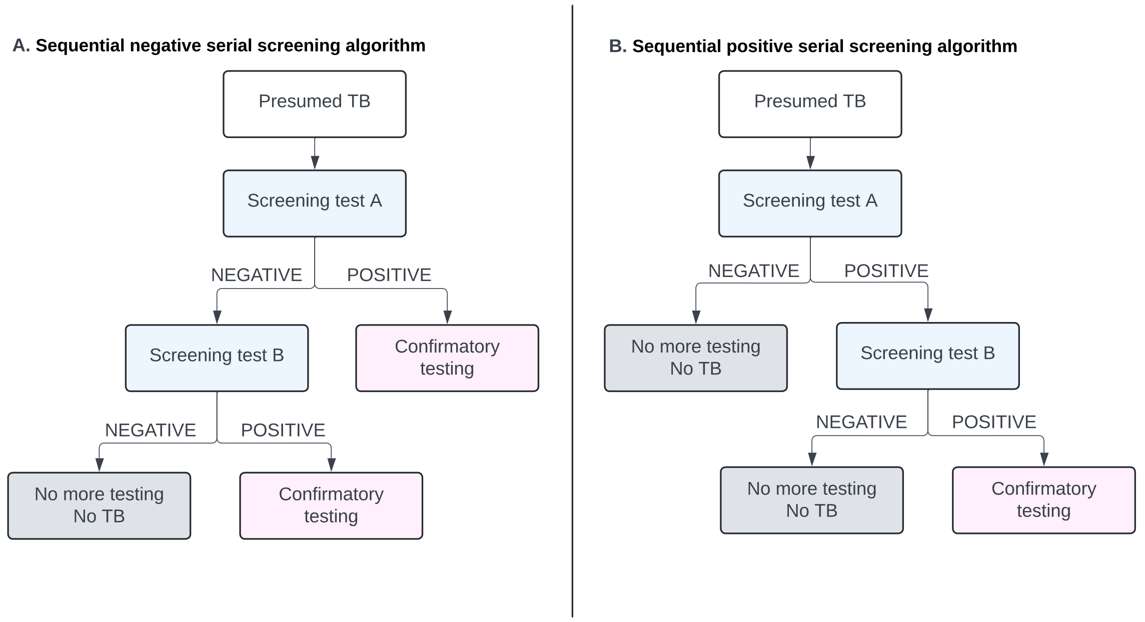

Figure S1. Sequential testing algorithms.
For each potential combination of screening tests, we considered a sequential negative serial screening approach (panel A) and a sequential positive serial screening approach (panel B).

Table S2. Summary of index test results by reference standard classification

**Microbiologic Reference Standard**		
**Index test, mean (SD)**	**Positive (n=303)**	**Negative (n=1,089)**
CRP	61.0 (65.3)	15.6 (37.4)
Xpert HR	-2.67 (0.94)	-1.15 (0.70)
CAD4TB	68.2 (26.0)	20.1 (23.2)

**Sputum Xpert Reference Standard**			
**Index test, mean (SD)**	**Positive (n=274)**	**Negative (n=1,114)**	**Indeterminate (n=4)**
CRP	65.3 (65.9)	15.6 (37.5)	43.6 (36.2)
Xpert HR	-2.79 (0.88)	-1.16 (0.70)	-2.19 (1.13)
CAD4TB	69.6 (25.1)	20.9 (24.0)	46.1 (39.2)

SD: standard deviation

Table S3. Results using subgroup-specific cut-points

	Using Philippines-specific cut-points (N=326 people in the Philippines, 29 [9%] with TB)		Using female-specific cut-points (N=623 females, 95 [15.3%] with TB)	
	Quantitative value indicating positive test	Specificity (95% CI)	Quantitative value indicating positive test	Specificity (95% CI)
**One-step screening**				
**CAD4TB**	TB score ≥7.1697	61.6% (55.8, 67.2)	TB score≥6.005	53.7% (49.4, 58.0)
**Xpert HR**	TB score ≤-0.8499	45.5% (39.7, 51.3)	TB score≤1.15	54.1% (49.8, 58.4)
**CRP**	Did not achieve 90% sensitivity		Did not achieve 90% sensitivity	
**Two-step screening using either test positive approach**				
**Xpert HR-CAD4TB**	Xpert HR ≤-2.69 or CAD4TB score ≥6.73	60.3% (54.5, 65.9)	Xpert HR ≤-1.318 or CAD4TB score ≥62.393	63.8% (59.6, 67.9)
**CRP-CAD4TB**	CRP≥10.79 or CAD4TB score≥10.49	65.0% (59.3, 70.4)	CRP≥36.105 or CAD4TB score≥10.988	61.9% (57.7, 66.1)
**CRP-Xpert HR**	CRP≥23.22 or Xpert HR≤-0.81	45.5% (39.7, 51.3)	CRP≥23.224 or Xpert HR≤-1.116	53.7% (49.4, 58.0)
**Two-step screening using both test positive approach**				
**Xpert HR-CAD4TB***	Xpert HR≤-0.658 and CAD4TB≥6.727	71.4% (65.9, 76.5)	Xpert HR≤-1.015 and CAD4TB≥3.079	63.2% (59.0, 67.3)
**CRP-CAD4TB**	CRP has no added value	-	-	-
**CRP-Xpert HR**	CRP has no added value	-	-	-

CI: confidence interval; CAD: computer aided detection; CRP: C-reactive protein; HR: host response; TPP: target product profile

* Meets current TPP target in the Philippines (≥90% sensitivity, ≥70% specificity)

^ Meets current TPP target among females (≥90% sensitivity, ≥70% specificity)

Table S4. Positive predictive value and negative predictive value in a hypothetical cohort of 1,000 with 10% TB prevalence

	Positive predictive value (95% CI) n=100 people with TB	Negative predictive value (95% CI) (n=900 people without TB)
**One-step screening**		
CAD4TB	25.2% (20.8, 30.0)	98.4% (97.2, 99.3)
Xpert HR	22.3% (18.3, 26.7)	98.3% (96.9, 99.2)
CRP	16.6% (13.5, 20.0)	97.8% (96.0, 98.9)
**Two-step screening using either test positive approach**		
Xpert HR-CAD4TB*	32.8% (27.3, 38.8)	98.6% (97.5, 99.3)
CRP-CAD4TB*	29.3% (24.3, 34.8)	98.6% (97.4, 99.3)
CRP-Xpert HR	21.8% (17.9, 26.2)	98.3% (96.9, 99.2)
**Two-step screening using both test positive approach**		
Xpert HR-CAD4TB*	27.5% (22.8, 32.7)	98.5% (97.3, 99.3)

CI: confidence interval

Table S5. The potential number of sputum tests averted by using a triage test in a hypothetical cohort of 1000 with 10% TB prevalence

	**Number that have first test performed** (total population)	**Number that have second test performed**	**Number of people with TB missed** (n=100 with TB)	**Number of people correctly classified by triage test**	**Number of people triage test positive, requiring sputum testing** (N=1000 with presumptive TB)	**Number of sputum tests averted** (N=1000 with presumptive TB)
**One-step screening**						
CAD4TB	1,000	0	10 (10%)	723 (72.3%)	357 (35.7%)	643
Xpert HR	1,000	0	10 (10%)	676 (67.6%)	404 (40.4%)	596
CRP	1,000	0	10 (10%)	537 (53.7%)	543 (54.3%)	457
**Two-step screening using either test positive approach**						
Xpert HR-CAD4TB*<br>CAD4TB-Xpert HR*	1,000	765<br>728	10 (10%)	806 (80.6%)	274 (27.4%)	726
CRP-CAD4TB*<br>CAD4TB-CRP*	1,000	851<br>643	10 (10%)	773 (77.3%)	307 (30.7%)	693
CRP-Xpert HR<br>Xpert HR-CRP	1,000	884<br>541	10 (10%)	668 (66.8%)	412 (41.2%)	588
**Two-step screening using both test positive approach**						
Xpert HR-CAD4TB*<br>CAD4TB-Xpert HR*	1,000	712<br>463	10 (10%)	753 (75.3%)	327 (32.7%)	673
CAD4TB-CRP	1,000	435	10 (10%)	716 (71.6%)	364 (36.4%)	636
Xpert HR-CRP	1,000	476	10 (10%)	670 (67%)	410 (41%)	590

Note. The order of tests in two-step screening does not impact the accuracy.
* Meets current TPP target (≥90% sensitivity, ≥70% specificity)

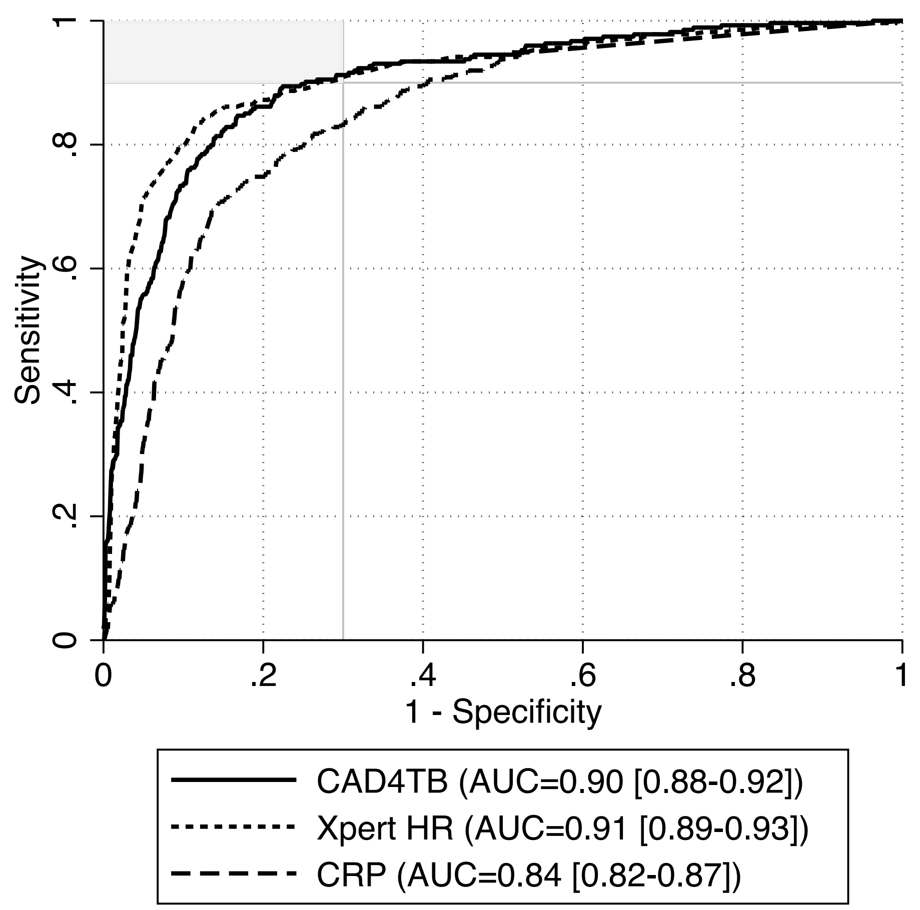

Figure S2. Sputum Xpert reference standard receiver operating characteristic curve. ROC curves with AUC and 95% CI displayed for CAD4TB, Xpert HR, and CRP. The upper-left area shaded in gray notes the region where tests meet TPP targets (≥90% sensitivity, ≥70% specificity). N=1,388 participants from the Philippines, Vietnam, Uganda, South Africa, and India with presumptive TB (n=274, 20% with sputum Xpert-positive TB). 4 participants included in the primary analysis had an indeterminate sputum Xpert Ultra result and were excluded from this analysis.

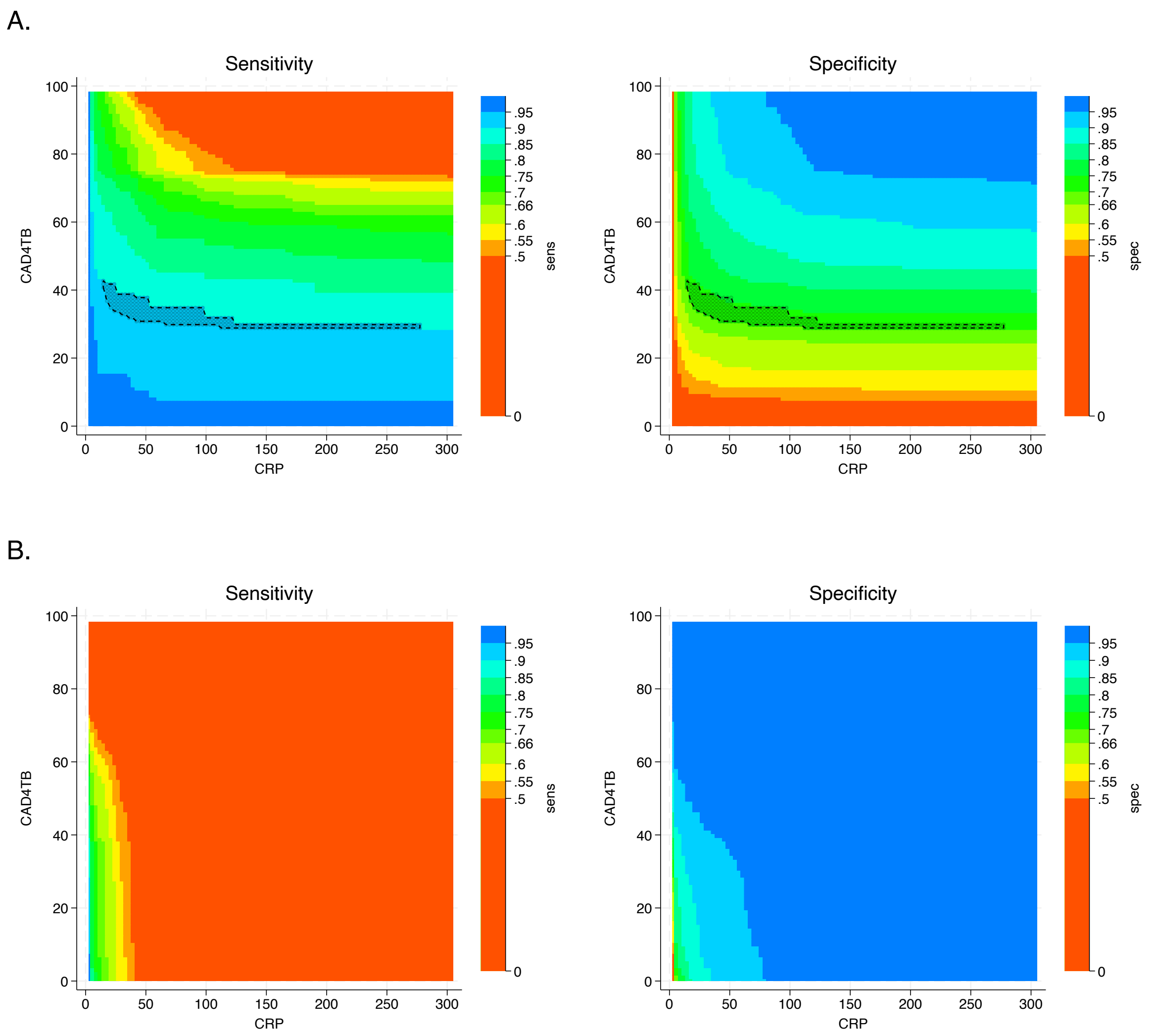

Figure S3. Selection of cut points for two-step screening algorithm combining CRP and CAD4TB. Panel (A) shows the possible cut-points using the sequential negative serial screening approach, in which the second screening test is conducted only if the first is negative and a positive screen is defined as positive on either test. Panel (B) shows the possible cut-points using the sequential positive serial screening approach, in which the second screening test is conducted only if the first is positive and a positive screen is defined as positive on both tests. The x-axis shows all potential cut-points for CAD4TB (test positive defined as greater than or equal to the cut point chosen), and the y-axis shows all potential cut points for CRP (test positive defined as greater than or equal to the cut point chosen). Each point on the graph corresponds to a pair of cut points used to define a positive screening algorithm. The colors represent the range of sensitivities and specificities possible. The outlined region contains pairs with sensitivity≥90% and specificity ≥70% (n=310 in panel A, n=0 in panel B).

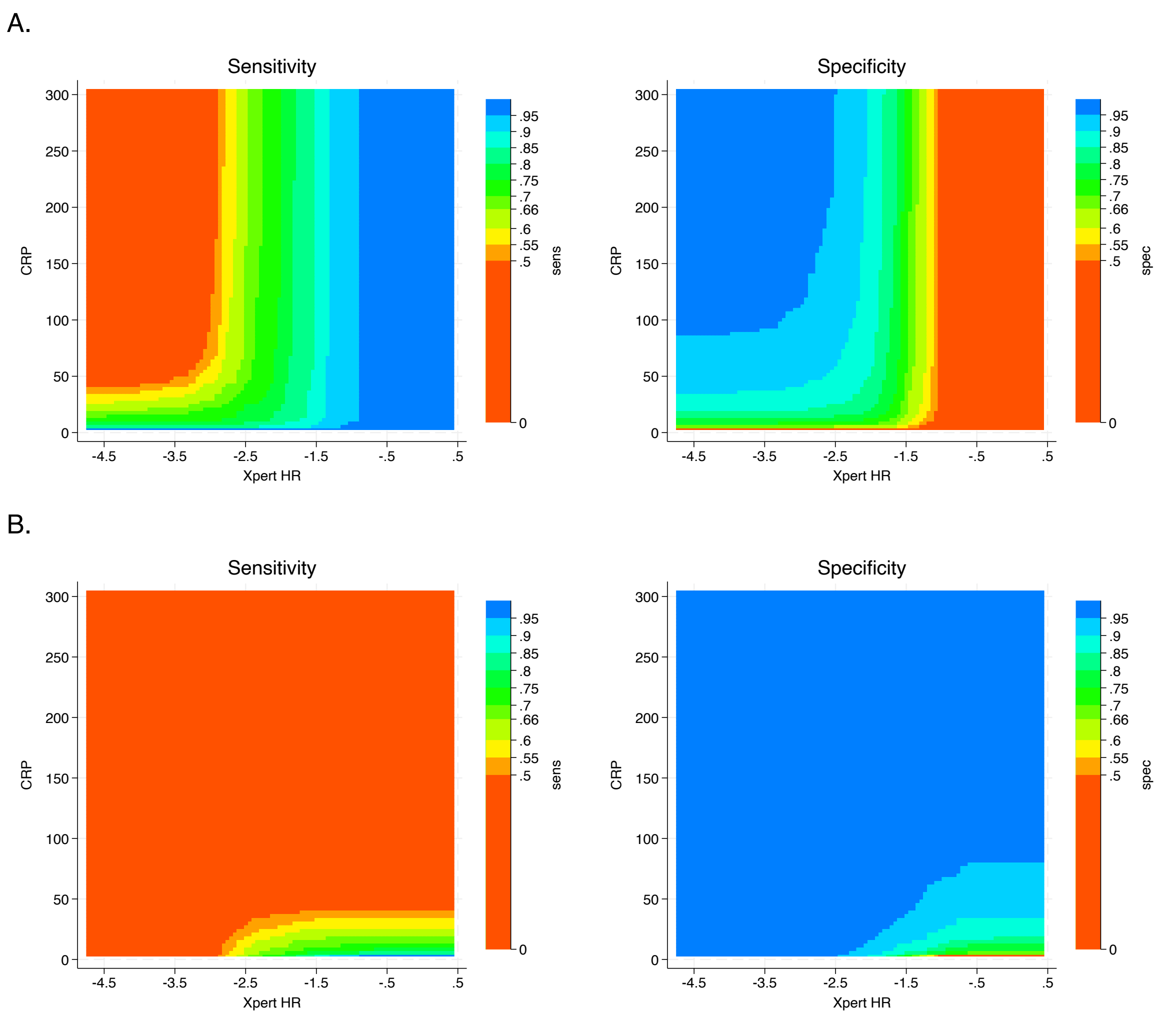

Figure S4. Selection of cut points for two-step screening algorithm combining CRP and Xpert HR. Panel (A) shows the possible cut-points using the sequential negative serial screening approach, in which the second screening test is conducted only if the first is negative and a positive screen is defined as positive on either test. Panel (B) shows the possible cut-points using the sequential positive serial screening approach, in which the second screening test is conducted only if the first is positive and a positive screen is defined as positive on both tests. The x-axis shows all potential cut-points for Xpert HR (test positive defined as less than or equal to the cut point chosen), and the y-axis shows all potential cut points for CRP (test positive defined as greater than or equal to the cut point chosen). Each point on the graph corresponds to a pair of cut points used to define a positive screening algorithm. The colors represent the range of sensitivities and specificities possible. No pair of cut points using either approach achieved sensitivity≥90% and specificity ≥70%.

Table S6. Head-to-head comparison of diagnostic accuracy against sputum Xpert reference standard. Cut points were chosen to achieve ≥90% sensitivity and maximize specificity against the sputum Xpert reference standard.

N=1,388	Quantitative value indicating positive test	Specificity (95% CI)	Absolute difference in specificity (95% CI)		
			vs. CAD4TB	vs. Xpert HR	vs. CRP
**One-step screening**					
**CAD4TB***	TB score ≥34.08	74.7% (72.0, 77.2)	-	1.7 (-1.7, 5.1)	15.4 (11.9, 18.8)
**Xpert HR***	TB score ≤-1.499	73.0% (70.3, 75.6)	-1.7 (-5.1, 1.7)	-	13.6% (10.3, 17.0)
**CRP**	≥4.07 mg/L	59.3% (56.4, 62.2)	-15.4 (-18.8, -11.9)	-13.6% (-17.0, -10.3)	-
**Two-step screening using either test positive appraoch**					
**Xpert HR-CAD4TB***	CAD4TB ≥62.59 or Xpert HR ≤-2.28	85.1% (82.9, 87.1)	10.4 (7.9, 12.9)	12.1 (9.4, 14.9)	25.8 (22.6, 29.0)
**CRP-CAD4TB***	CRP ≥45.71 mg/L or CAD4TB ≥39.16	78.0% (75.5, 80.4)	3.3 (1.3, 5.3)	5.0 (1.8, 8.3)	18.7 (15.5, 21.8)
**CRP-Xpert HR***	CRP ≥265.23mg/L or Xpert HR ≤-1.495	72.9% (70.2, 75.5)	-1.8 (-5.2, 1.6)	0.1 (-0.4, 0.2)	13.6 (10.2, 16.9)
**Two-step screening using both test positive approach**					
**Xpert HR-CAD4TB***	CAD4TB ≥31.82 and Xpert HR ≤-0.76	77.6% (75.1, 80.1)	3.0 (1.6, 4.3)	4.7 (1.5, 7.9)	18.3 (15.0, 21.6)
**CRP-CAD4TB**	CRP has no added value	-	-	-	-
**CRP-Xpert HR**	CRP has no added value	-	-	-	-

CI: confidence interval; CAD: computer aided detection, represented by CAD4TB; CRP: C-reactive protein; HR: host response; TPP: target product profile

N=1,392 people with presumptive TB, n=274 with sputum Xpert Ultra positive TB
Note. 4 participants included in the primary analysis had an indeterminate sputum Xpert Ultra result and were excluded from this analysis.
* Meets current TPP target (≥90% sensitivity, ≥70% specificity)

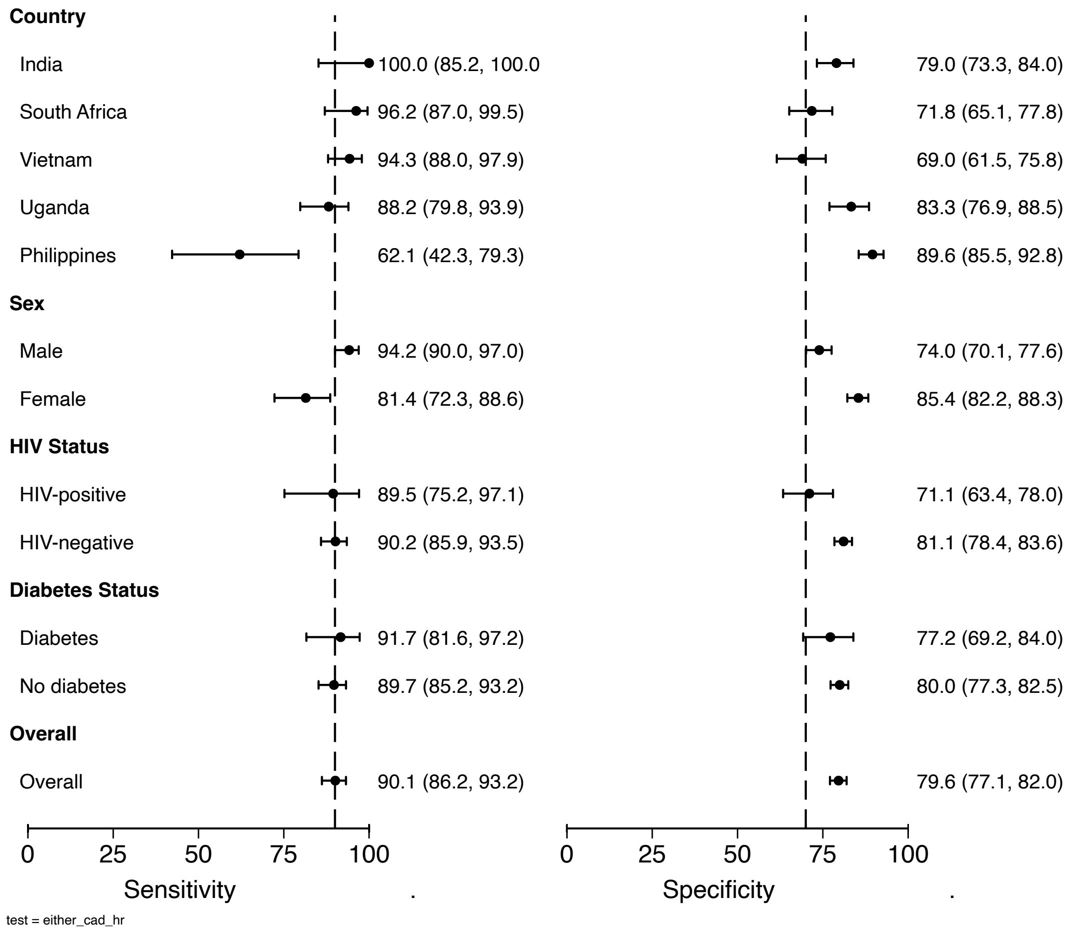

Figure S5. Subgroup analysis of two-step screening algorithm Xpert HR-CAD4TB using a sequential negative serial screening algorithm.

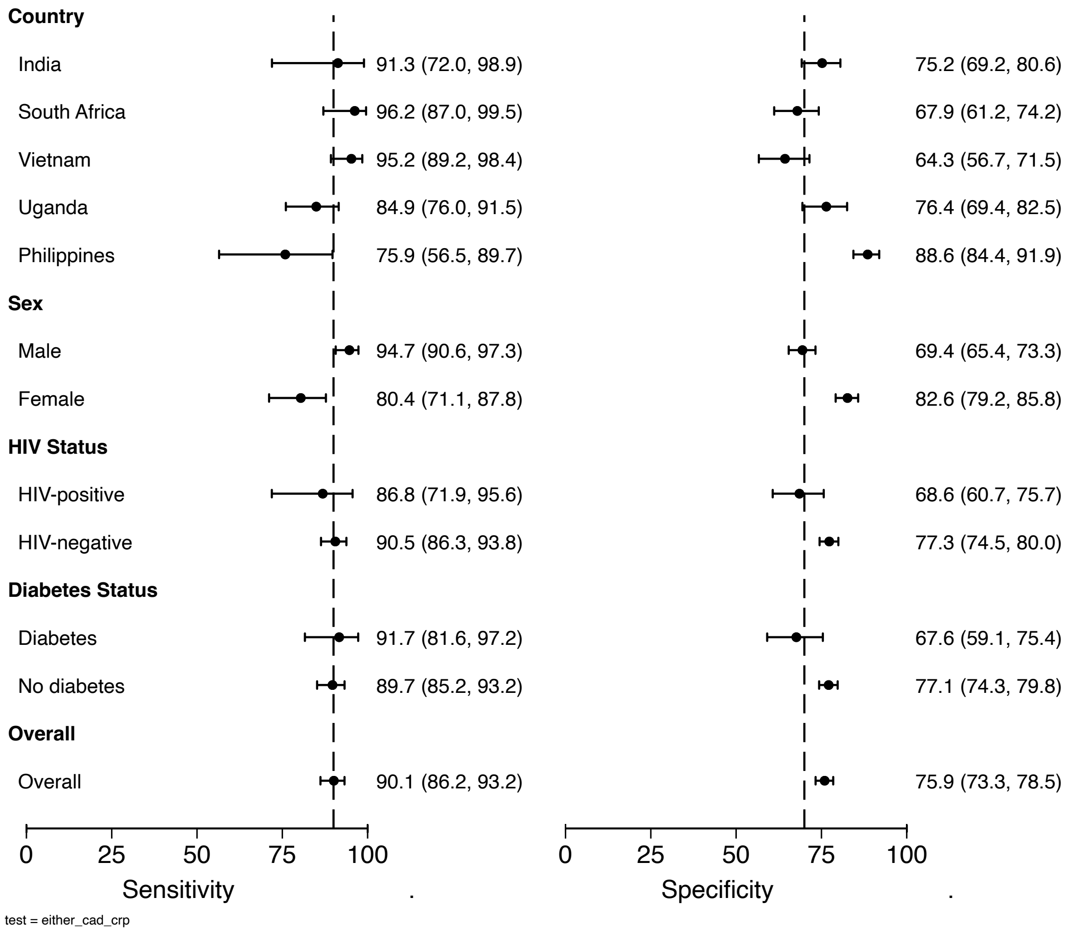

Figure S6. Subgroup analysis of two-step screening algorithm CRP-CAD4TB using a sequential negative serial screening algorithm.

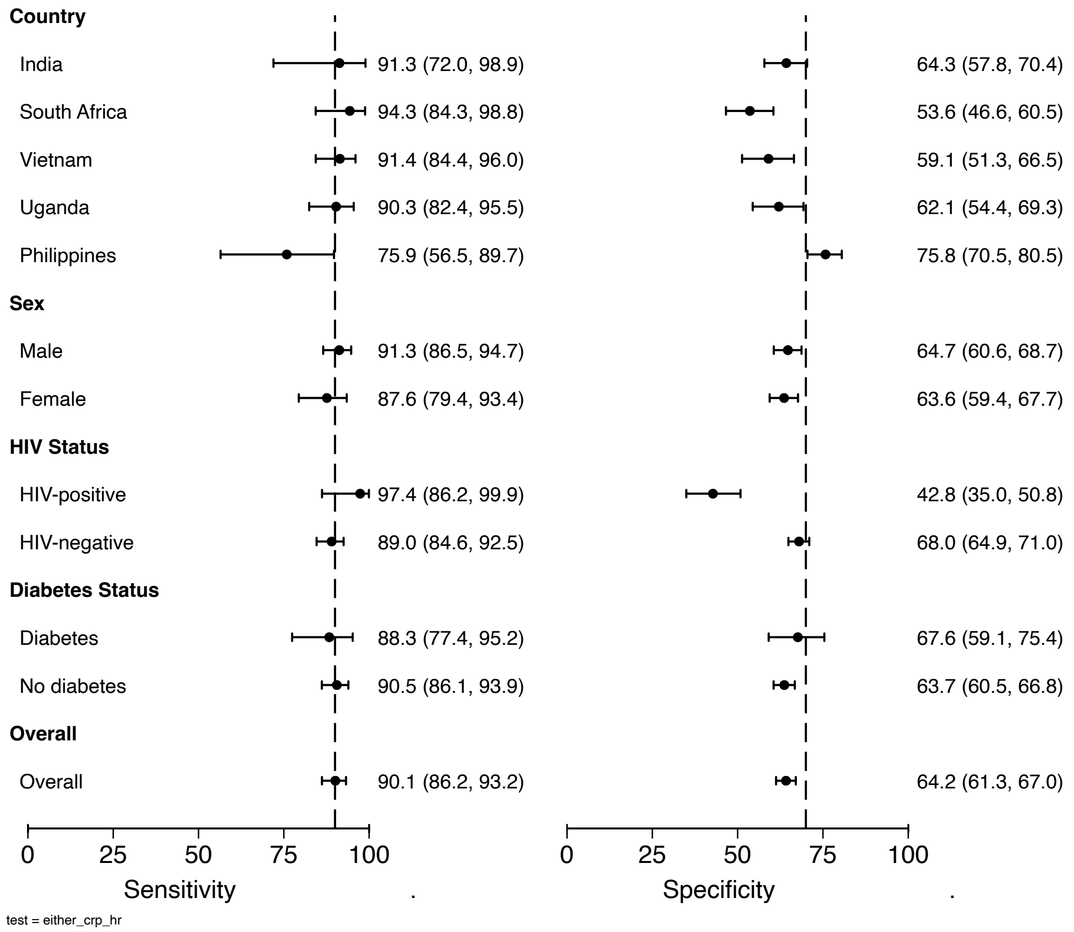

Figure S7. Subgroup analysis of two-step screening algorithm CRP-Xpert HR using a sequential negative serial screening algorithm.

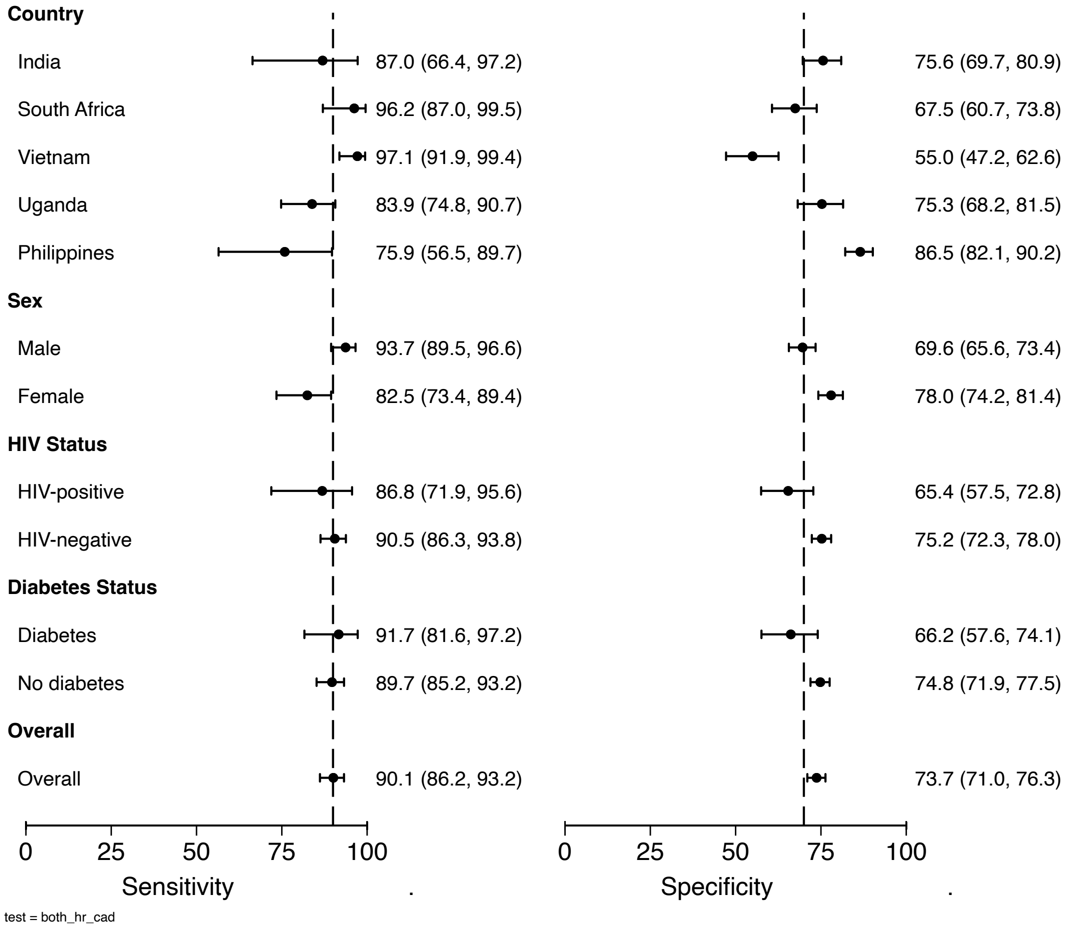

Figure S8. Subgroup analysis of two-step screening algorithm Xpert HR-CAD using a sequential negative serial screening algorithm.

Table S7. Agreement between binary triage tests (percent agreement, Cohen's Kappa statistic)

Percent agreement (Kappa)	CAD4TB	Xpert HR	CRP		CAD-Xpert HR (sequential negative)	CAD-CRP (sequential negative)	CRP-Xpert HR (sequential negative)		CAD-Xpert HR (sequential positive)	CAD-CRP (sequential positive)	CRP-Xpert HR (sequential positive)
**CAD4TB**	-	69.0% (0.38)	65.5% (0.33)		83.3% (0.65)	92.0% (0.84)	70.0% (0.40)		92.0% (0.84)	99.4% (0.99)	69.0% (0.37)
**Xpert HR**	-	-	69.4% (0.39)		76.2% (0.51)	71.8% (0.43)	97.4% (0.95)		75.1% (0.50)	69.4% (0.38)	99.4% (0.99)
**CRP**	-	-	-		66.1% (0.36)	66.8% (0.36)	71.1% (0.43)		67.0% (0.36)	65.8% (0.33)	69.8% (0.40)
**CAD-Xpert HR (sequential negative)**	-	-	-		-	88.5% (0.75)	77.0% (0.53)		83.5% (0.65)	82.9% (0.64)	75.7% (0.51)
**CAD-CRP (sequential negative)**	-	-	-		-	-	73.9% (0.47)		88.9% (0.77)	91.8% (0.83)	71.6% (0.43)
**CRP-Xpert HR (sequential negative)**	-	-	-		-	-	-		75.7% (0.51)	70.6% (0.41)	97.1% (0.94)
**CAD-Xpert HR (sequential positive)**	-	-	-		-	-	-		-	92.5% (0.85)	75.1% (0.50)
**CAD-CRP (sequential positive)**	-	-	-		-	-	-		-	-	69.3% (0.38)
**CRP-Xpert HR**	-	-	-		-	-	-		-	-	-

**(sequential positive)**											
